# ASL and susceptibility-weighted imaging contribution to the management of acute ischaemic stroke

**DOI:** 10.1007/s13244-016-0529-y

**Published:** 2016-11-07

**Authors:** Sébastien Verclytte, Olivier Fisch, Lucie Colas, Olivier Vanaerde, Manuel Toledano, Jean-François Budzik

**Affiliations:** 1 Imaging Department, Lille Catholic Hospitals, Lille, France; 2 Lille, France, Lille Catholic University, Lille, France

**Keywords:** Susceptibility-weighted imaging, Arterial spin labelling, Perfusion, Stroke, MRI

## Abstract

**Abstract:**

Magnetic resonance imaging (MRI) plays a central role in the early diagnosis of cerebral vascular events. Today, MRI is used not only for the detection of acute ischaemic lesions, but also to fine tune the diagnosis and improve patient selection for early therapeutic decision-making. In this perspective, new tools such as arterial spin labelling (ASL) and susceptibility-weighted imaging (SWI) sequences have been developed. These MRI sequences enable noninvasive assessment of brain damage, providing important diagnostic and prognostic information: evaluation of cerebral parenchymal perfusion; detection and aetiological assessment of thrombi; ruling out differential diagnoses. After a brief recall of the fundamental basis of these sequences, this article proposes an update on their current contribution to the early management of stroke victims.

***Teaching Points*:**

• *These noninvasive sequences provide essential information for early management of acute stroke.*

• *They can detect zones of parenchymal hypoperfusion.*

• *Susceptibility-weighted sequences provide information on thrombus localisation and composition.*

• *ASL can identify certain aetiologies of stroke mimics.*

• *Post-therapeutic ASL perfusion status predicts outcome.*

## Introduction

MRI is an advanced tool for pre-therapeutic management of acute stroke. MRI can be used to assess the extent of brain infarction, localise the site of arterial occlusion, and search for evidence ruling out potential contraindications for thrombolysis. In recent years, the advent of 3-T MRI scanners for routine clinical applications has incited interest in new sequences exploiting the higher field strength, e.g. arterial spin labelling (ASL) and susceptibility-weighted imaging (SWI) sequences. This opens the way for new perspectives such as noninvasive assessment of parenchymal hypoperfusion, precise localisation of the thrombus and its origin, or characterisation of nonvascular stroke mimics. Here we propose an update on the contribution of these new techniques for acute-phase management of stroke patients.

## SWI sequences

### Fundamentals

These gradient-echo sequences are acquired with a long echo time (TE) in order to take full advantage of the magnetic susceptibility phenomenon. Magnetic susceptibility corresponds to the variation in the local magnetic field of a material exposed to an external magnetic field. This occurs for instance in the venous compartment, which contains a large amount of deoxyhaemoglobin, a highly paramagnetic substance. Paramagnetic substances create a field oriented in the same direction as the higher intensity main field, leading to a lower local signal. When a long TE is used, the dephasing resulting from spin-spin interactions and field heterogeneity is increased. TE can thus be set to yield a phase opposition phenomenon between deoxyhaemoglobin and the adjacent parenchyma, further lowering the signal [1]. Moreover, magnetic susceptibility evolves proportionally with the B0 magnetic field so that the phenomenon is more marked with 3 T than 1.5 T.

There are several types of susceptibility-weighted sequences. Susceptibility-weighted imaging (Siemens Heathcare, Erlangen, Germany) and Venobold (Philips Healthcare, Best, The Netherlands) are based exclusively on reading a long TE. Other sequences, such as susceptibility-weighted angiography (SWAN) (General Electrics Healthcare, Milwaukee, WI, USA) and susceptibility-weighted imaging with phase enhancement (SWIp) (Philips Healthcare, Best, The Netherlands), are based on reading multiple TEs set at long and short values. This method takes advantage of the more marked time-of-flight (TOF) effect when reading shorter TEs, adding to the magnetic susceptibility effect observed on longer TE images.

### Applications for pre-therapeutic management of acute ischaemic stroke

#### Haemorrhagic transformation

Susceptibility-weighted sequences are much more sensitive for the detection of haemorrhagic transformation than either non-contrast CT scan or T2 gradient echo sequences. This greater sensitivity is important not only in the acute phase of ischaemic stroke, but is also highly contributive to the diagnosis of all types of intracranial bleeding [2–5].

#### Arterial thrombus

One of the major challenges for MRI exploration of acute stroke is to search for aetiological elements and factors predictive of post-therapeutic outcome. SWI sequences provide information on thrombus localisation and composition.

Intra-arterial signal voids on T2 gradient echo images, termed susceptibility vessel sign, were initially described as suggestive of cardioembolic thrombi [6, 7]. Indeed, those thrombi are mainly composed of red cells and thus blood degradation products have a strong paramagnetic effect compared with fibrin-rich atheromatous thrombi [8].

**Fig. 1:**
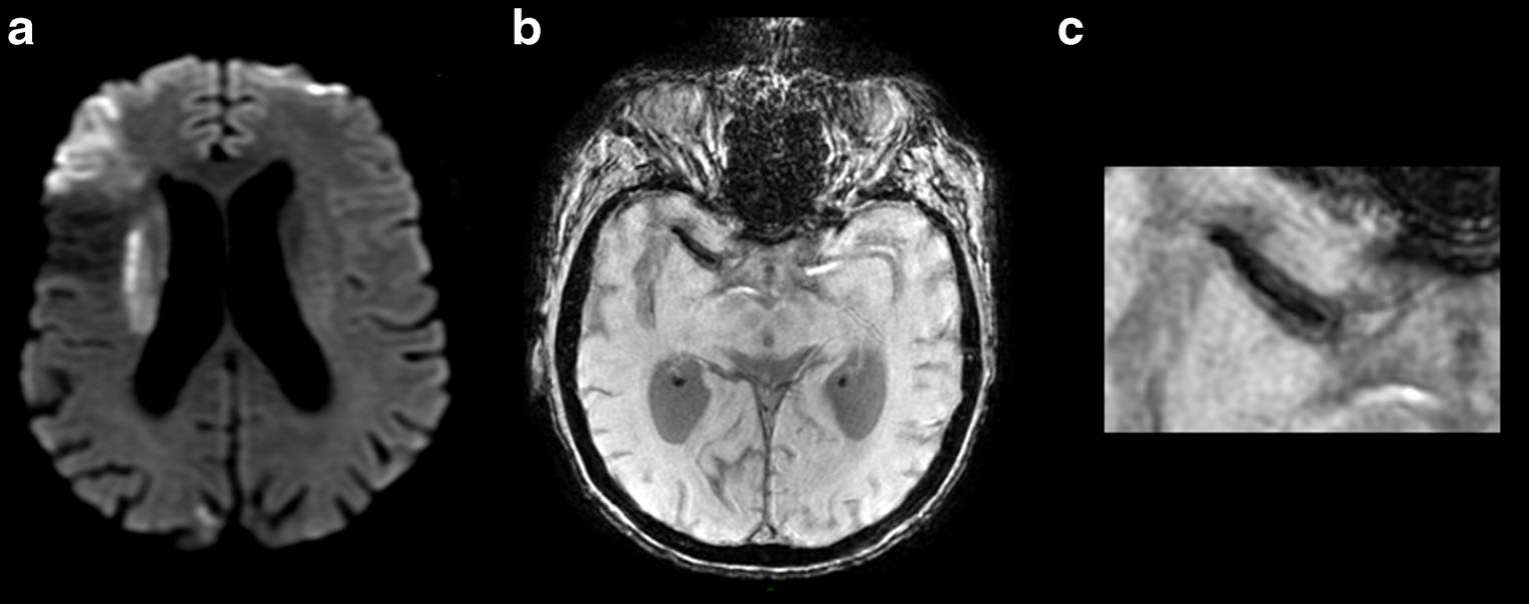
An 85-year-old patient presenting left hemibody deficit on DWI (**a**) and SWI (**b** and **c**) sequences in the axial plane. **a** Acute superficial sylvian and deep right ischaemic event. **b** and **c** Long thrombus in the M1 segment of the right middle cerebral artery with a two-layered susceptibility sign

It appears that a more recently described two-layered susceptibility sign would be more sensitive and much more specific for cardioembolic thrombi than the susceptibility vessel sign (Fig. 1), which can arise via many mechanisms [9].

Whatever the origin of the thrombus, in acute ischaemic stroke, the susceptibility vessel sign would be correlated with:

- a lower rate of recanalisation after intravenous thrombolysis compared with arterial occlusion without the susceptibility vessel sign [10, 11], particularly for proximal localisations [12], for lengths greater than 20 mm, for thrombi with irregular contours [13], and for susceptibility artefacts extending beyond the arterial lumen [14];
- a favourable 3-month functional outcome in patients who undergo mechanical thrombectomy for anterior circulation occlusion [15], but not with a higher rate of recanalisation [16].

**Fig. 2:**
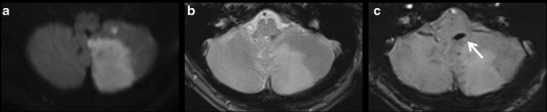
A 48-year-old patient presenting sudden-onset vertigo. DWI (**a**), T2 spin-echo (**b**), and SWI (**c**) sequences in the axial plane. **a** Acute ischaemic lesion in the territory of the left posterior inferior cerebellar artery. **b** No intra-vascular signal anomaly. **c** Susceptibility vessel sign revealing an intra-arterial thrombus in the left posterior inferior cerebellar artery

**Fig. 3:**
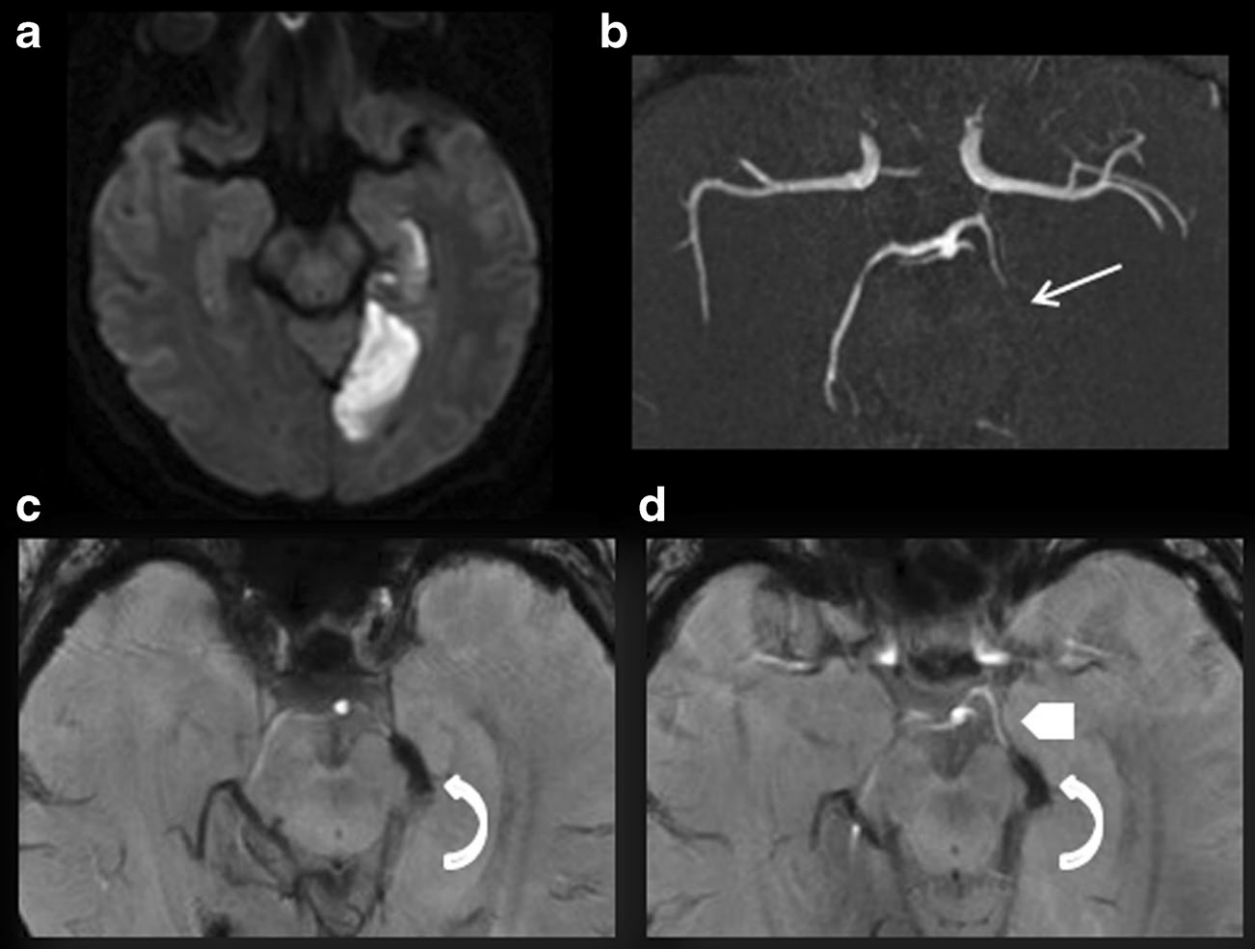
A 72-year-old patient with right homonymous lateral hemianopsia. DWI (**a**), 3D TOF (**b**), minimum intensity projection (**c**), and multiplanar reconstruction (**d**) of the SWAN sequence in the axial plane. **a** Acute ischaemic lesion in the territory of the left posterior cerebral artery. **b** No visualisation of the left P2 (*white arrow*). **c** and **d** Susceptibility vessel sign in P2 (*curved arrow*). **d** TOF effect of the SWAN sequence identifies the susceptibility vessel sign associated with the thrombus and the upstream arterial segment (arrowhead)

Because of its greater sensitivity, and particularly so with a strong magnetic field, SWI offers a more precise assessment of thrombus morphology. For determining the site of occlusion, SWI exhibits better sensitivity and specificity than T2 gradient echo (Fig. 2) or 3D TOF imaging [17–19]. It is also much more effective in identifying distal thrombi, for both anterior [17, 20, 21] and posterior [19] localisations (Fig. 3). Detection of multiple distal thrombi is of major importance, since in this situation the 3-month functional outcome is less favourable compared with a unique occlusion [22]. However, distal thrombi may be confused with hypointense venous structures or microbleeds on SWI. The sequences based on a multi-TE readout are more efficient in doubtful cases. Indeed, the TOF effect related to the shortest TE read allows confirming the intra-arterial origin of the signal void assigned to the thrombus thanks to the susceptibility effect.

#### Brush sign

**Fig. 4:**
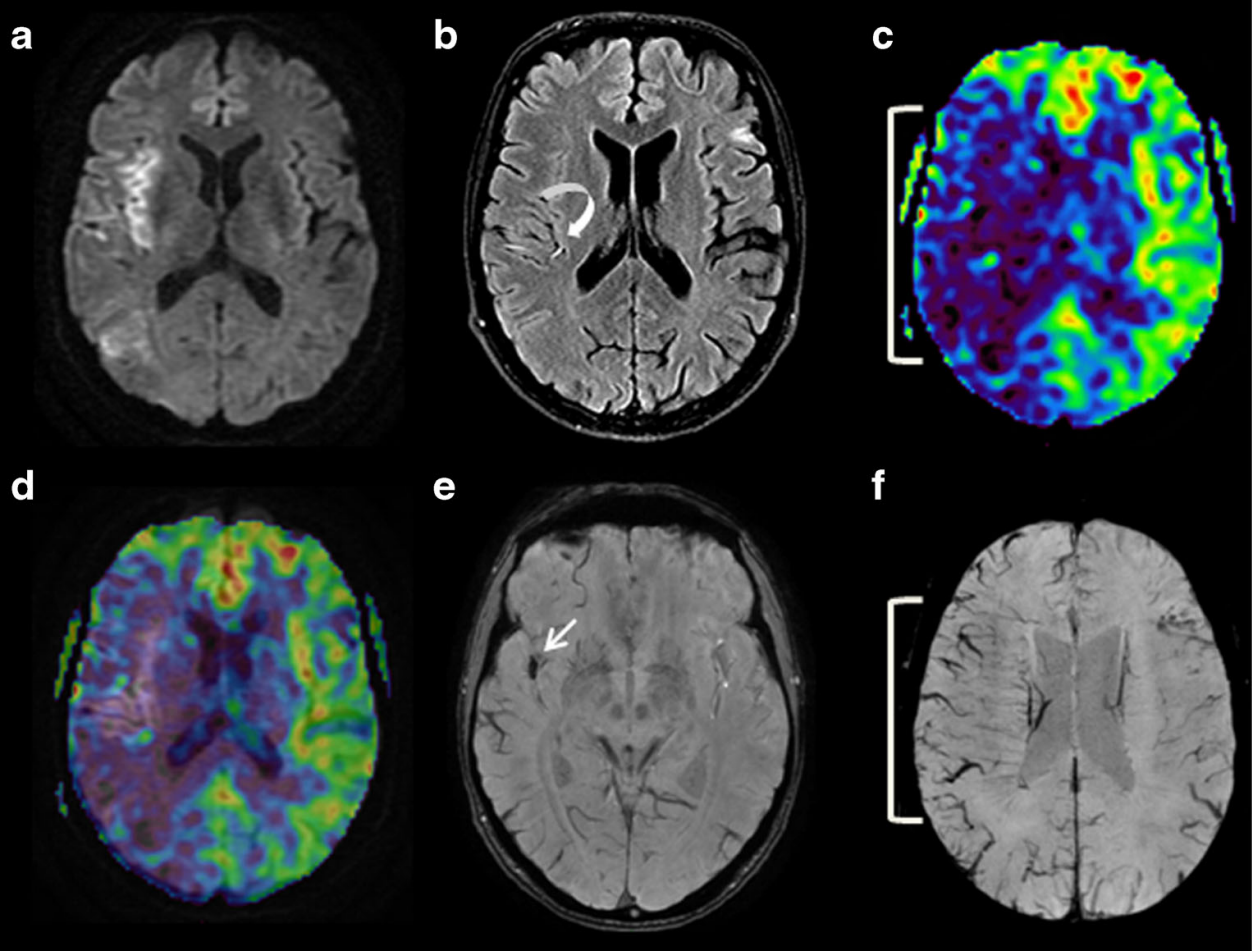
A 76-year-old patient seen in an emergency setting for right brachiofacial motor deficit 3 h after symptom onset. DWI (**a**), FLAIR (**b**), ASL (**c**), ASL/DWI fusion (**d**), and SWI (**e** and **f**) sequences in the axial plane. **a** Acute right superficial sylvian infarction. **b** The FLAIR sequence fails to visualise any infarct zone. Slow circulation in the cortical branch of the right middle cerebral artery, hypersignal (*curved arrow*). **c** Blue zone (*white parentheses*) visualises a wide right sylvian zone of hypoperfusion. **d** Mismatch: DWI hypersignal and ASL hypoperfusion. **e** Susceptibility vessel sign in the M2 segment of the right middle cerebral artery, thrombus. **f** Right sylvian (*white parentheses*) brush sign; the extension is the same as the hypoperfusion zone visible on the ASL sequence (image **c**)

Thanks to the BOLD effect, SWI sequences can also be used to indirectly assess deoxyhaemoglobin content in peri-encephalic veins. Indeed, when exposed to experimental hypoxia, the venous compartment gives a proportionally lower signal that can be detected visually [23]. During acute ischaemia, the local oxygen deprivation secondary to arterial occlusion is seen as a hypointense zone in the cortical and deep veins called the brush sign [24], as multiple hypointense vessels [25], or as prominent vessel [26]. The presence of these signs in the acute phase is associated with a less severe clinical presentation (lower initial NIHSS score), lower lesion diffusion-weighted imaging (DWI) volume, more extensive penumbra, and more pronounced collaterals [25]. Moreover, the brush sign, reflecting cerebral hypoperfusion, would be correlated with penumbra volume. Luo et al. [27] demonstrated the absence of significant mismatch between DWI-MTT (mean transit time) maps produced by the dynamic susceptibility contrast MRI (DSC-MRI) and DWI-SWI maps (Fig. 4). Susceptibility-weighted sequences would thus enable effective noninvasive measurement of the penumbra in acute ischaemic stroke.

In the absence of thrombolytic treatment, the initial extent of the brush sign would be correlated with the final infarct volume and the severity of the functional outcome [26]. In case of middle cerebral artery occlusion treated by intravenous thrombolysis, the presence of a brush sign would be associated with a higher risk of haemorrhagic transformation and a less favourable 3-month functional outcome [28].

## Arterial spin labelling

### Fundamentals

Arterial spin labelling (ASL) is a brain perfusion sequence that does not require contrast injection. A salve of radiofrequency waves is applied to a box positioned in the neck area, upstream from the brain region to be studied in order to locally saturate the proton spins of the water molecules in the arterial blood and thus play the role of an endogenous contrast agent. After a certain transit time, which depends upon the subject’s age and circulatory conditions, the saturated protons reach the brain parenchyma generating the labelled image. A second acquisition is made without prior saturation of the water molecule spins, generating a control image. Subtraction of the labelled and control images generates a perfusion-weighted image used to produce an absolute measurement of cerebral blood flow (CBF). There are several types of ASL sequences. Continuous ASL (CASL), pulsed ASL (PASL), and pseudo-continuous (pCASL) are based on different excitation methods and present specific advantages and disadvantages.

In general, the CASL method has a higher signal-to-noise (S/N) ratio but induces an excessive specific absorption rate, particularly at 3 T. With PASL, labelling is particularly effective, but with a low S/N ratio. The pCASL method has the advantages of both the preceding methods, with a satisfactory S/N ratio and a limited specific absorption rate. It is currently recommended for clinical applications, preferably with turbo spin-echo 3D acquisition [29].

Post-labelling delay (PLD) should to be optimal for pCASL. This parameter should be adjusted on a case-by-case basis and correspond as closely as possible to the time needed for labelled protons to reach the region of interest. If the PLD is too short, all of the labelled bolus may not have time to fully integrate the parenchyma to be explored, particularly junctional areas. This can lead to a significant local signal loss, which could be misdiagnosed as false hypoperfusion regions. Patient-related factors can also lead to systemised false hypoperfusion areas, resulting for example from stenosis of the supra-aortic trunks, which produces a longer transit time between the labelling zone and the region of interest. In this case, it may be difficult to differentiate between false and real hypoperfusion, and other sequences, such as DWI or MR angiography, may be helpful to confirm the diagnosis.

Other artefacts related to the arterial transit can also occur. Seen as linear or serpingious hypersignals within the arteries of the Willis polygon, they are related to the persistence of labelled protons in the vascular compartment because of an overly short PLD. PLD is thus an essential parameter that must be adjusted to the patient’s circulatory status.

Standardised PLD values have been validated for patient age and pathological condition: 1500 ms for children; 1800 ms for healthy adults aged <70 years, and 2000 ms for adults aged >70 years or for patients with a suspected neurological condition, irrespective of the origin [29].

It should be pointed out that ASL is sensitive to motion. It is recommended to use background suppression and prospective correction methods to reduce motion artefacts [29]. However, in cases of highly agitated or confused patients, good quality ASL maps remain difficult to obtain.

### Applications for the exploration of acute ischaemic stroke

#### Evaluation of the penumbra zone and DWI/perfusion mismatch

Several 3-T MRI studies have provided objective evidence of the good correlation among computed tomography perfusion, DSC-MRI, and ASL for determining zones of parenchymal hypoperfusion in acute stroke (Fig. 4) [30–33]. Thus ASL provides a reliable assessment of penumbra volume based on the following CBF values:

- ASL-CBF <20 ml/100 g/min. This is correlated with MTT >10 s on DSC-MRI [34];
- ASL-CBF 40 % lower than in healthy contralateral tissue. Lesion volumes thus determined are correlated with volumes measured on computed tomography perfusion maps (Tmax 5.5 s) and DSC-MRI (Tmax + 6 s) as well as with the 24-h DWI lesion in patients without reperfusion [30].

ASL reliability and reproducibility have been established for 3-T assessment of penumbra volume, while the lower S/N ratio hampers 1.5-T performance [35].

#### Localisation of the arterial thrombus

**Fig. 5:**
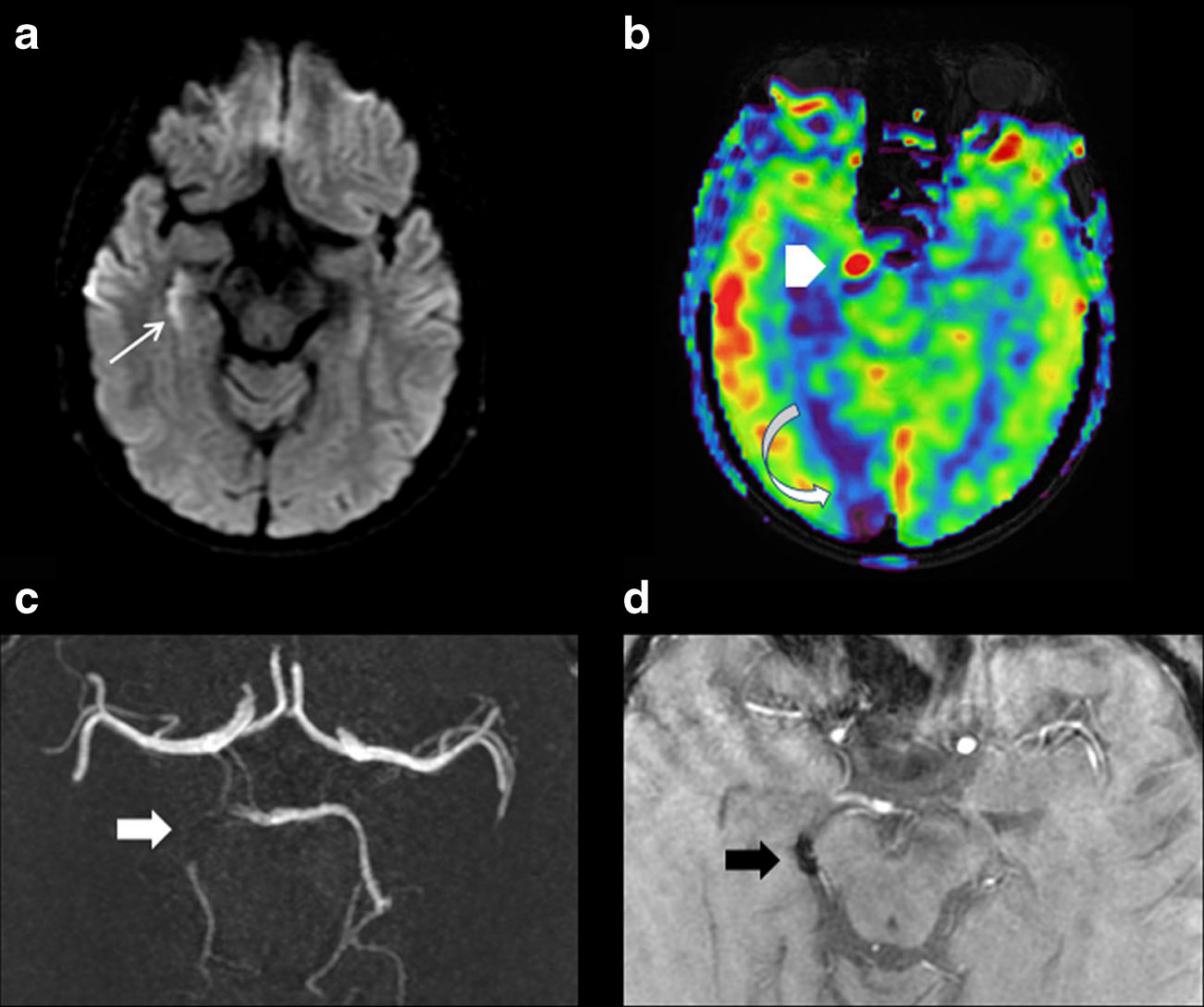
A 76-year-old patient seen in an emergency setting for sudden-onset left hemibody hypoesthesia. DWI (**a**), ASL (**b**), 3D TOF (**c**), and SWI (**d**) sequences in the axial plane. **a** Small infarct zone in the right internal temporal region. **b** Intravascular hypersignal; bright vessel sign upstream from the thrombus (*arrowhead*). Right occipital hypoperfusion with DWI mismatch (*curved arrow*). **c** Visualisation defect in the P2 segment of the right posterior cerebral artery (*white arrow*). **d** Susceptibility vessel sign in P2; thrombus (*black arrow*)

During the acute phase of ischaemic stroke, a bright vessel appearance on ASL sequences localises the thrombus (Fig. 5). This bright vessel sign corresponds to an accumulation of protons in labelled arterial blood immediately upstream from the arterial occlusion. The sensitivity of the bright vessel sign would be superior to that of the susceptibility vessel sign [36–38]. The bright vessel sign can also reveal certain distal arterial occlusions not initially detected on the vascular sequences, e.g. 3D TOF sequences [38].

#### Post-therapeutic hyperperfusion

When early arterial recanalisation occurs after intravenous thrombolysis, focal zones of hyperperfusion, termed luxury perfusion, can appear within the initial hypoperfusion zone. These zones are sometimes visible only on the ASL sequences and not on DSC-MRI, further complicating their interpretation [39–41].

**Fig. 6:**
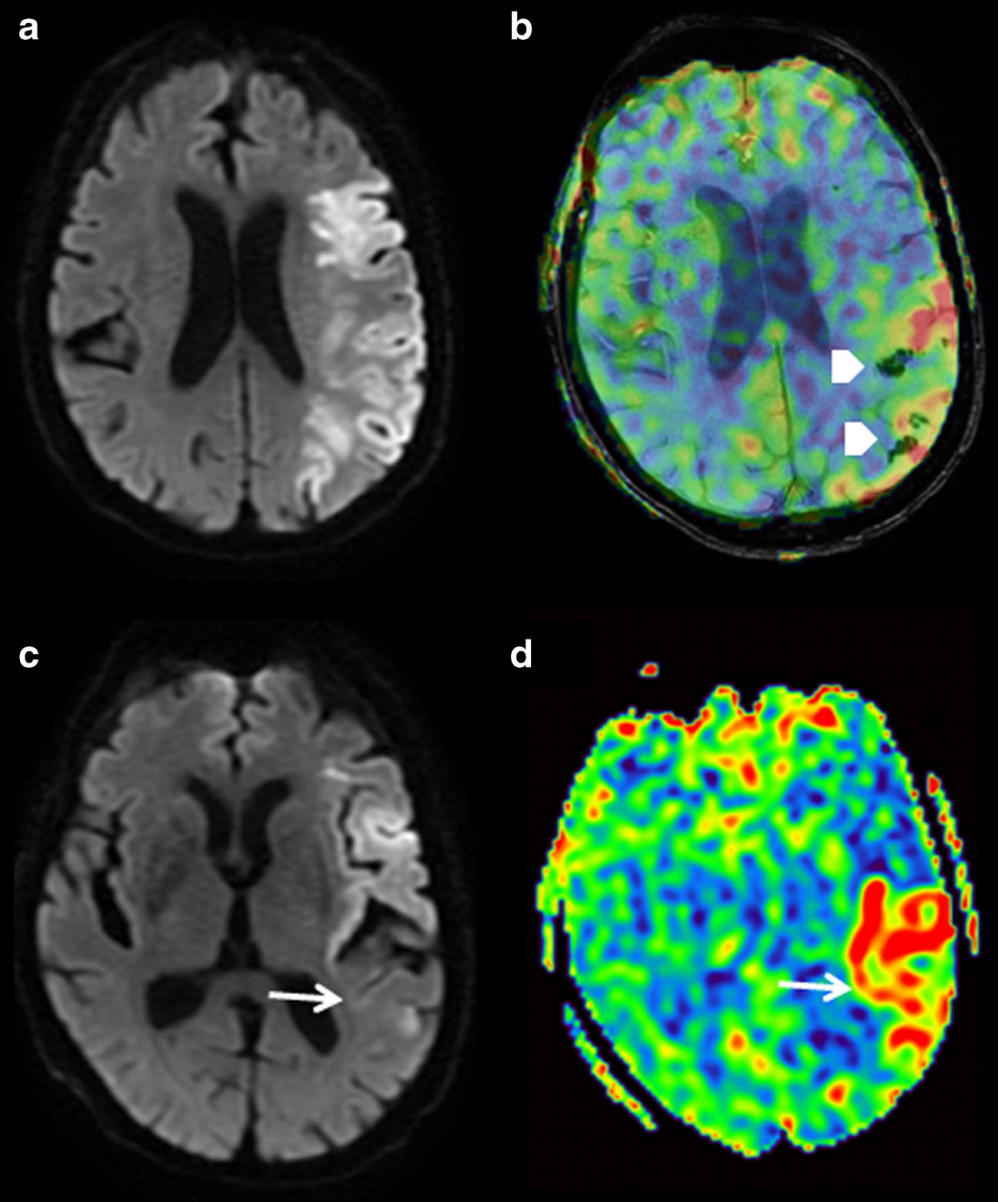
Control image 24 h after intravenous thrombolysis in a patient seen in an emergency setting for a superficial left sylvian ischaemia with favourable clinical outcome. DWI (**a** and **c**) SWI/ASL fusion (**b**), and ASL (**d**) sequences. **a** Left superficial sylvian acute ischaemic lesion. **b** Zones of hypointense haemorrhagic transformations on the SWI sequence superimpose with the hyperperfusion zones on the ASL (*arrowheads*). **c** and **d** Anterior sylvian involvement with partial recovery on the DWI images of the posterior portion corresponding to the zone of hyperperfusion (*white arrows*)

Thus the presence of hyperperfusion zones on the ASL sequences of a control MRI early after thrombolysis is associated with improved functional outcome at 24 h and 3 months and with a smaller final infarct volume [39, 40]. These zones of hyperperfusion would correspond to preserved regions that achieve *restitutio ad integrum* after the acute episode [40] (Fig. 6).

In opposition, there is ongoing debate on how early hyperperfusion zones would be associated with haemorrhagic risk since the available evidence is contradictory [40, 41]. Nevertheless, outcome would be better in hyperperfusion patients independently of the presence or not of haemorrhagic transformation [40]. Post-therapeutic ASL perfusion status predicts outcome.

#### Diagnosis of stroke mimics

Stroke mimics are non-vascular neurological pathologies that reproduce the symptoms of stroke. According to the literature, they occur in 1 to 14.5 % [42–48] of patients treated with intravenous thrombolysis for suspected acute stroke, with a mean of 4.38 % [46]. These different studies report that these patients have a low risk of haemorrhage, estimated at 0 to 1 %, and a better functional prognosis than patients who undergo thrombolysis for confirmed ischaemic stroke [49]. There is however a significant treatment-related cost increment, estimated at $5400 in one American study [49]. These elements should incite efforts to optimise candidate selection for thrombolysis.

In order of frequency, the causes of stroke mimics are: partial epilepsy and psychiatric disorders, and then at variable frequencies depending on the report, infectious diseases (meningitis and meningoencephalitis), migraine with aura, brain tumours, cortical vein thrombosis, demyelinating inflammatory diseases, and metabolic or toxic pathologies. ASL can identify certain aetiologies of stroke mimics.

**Fig. 7:**
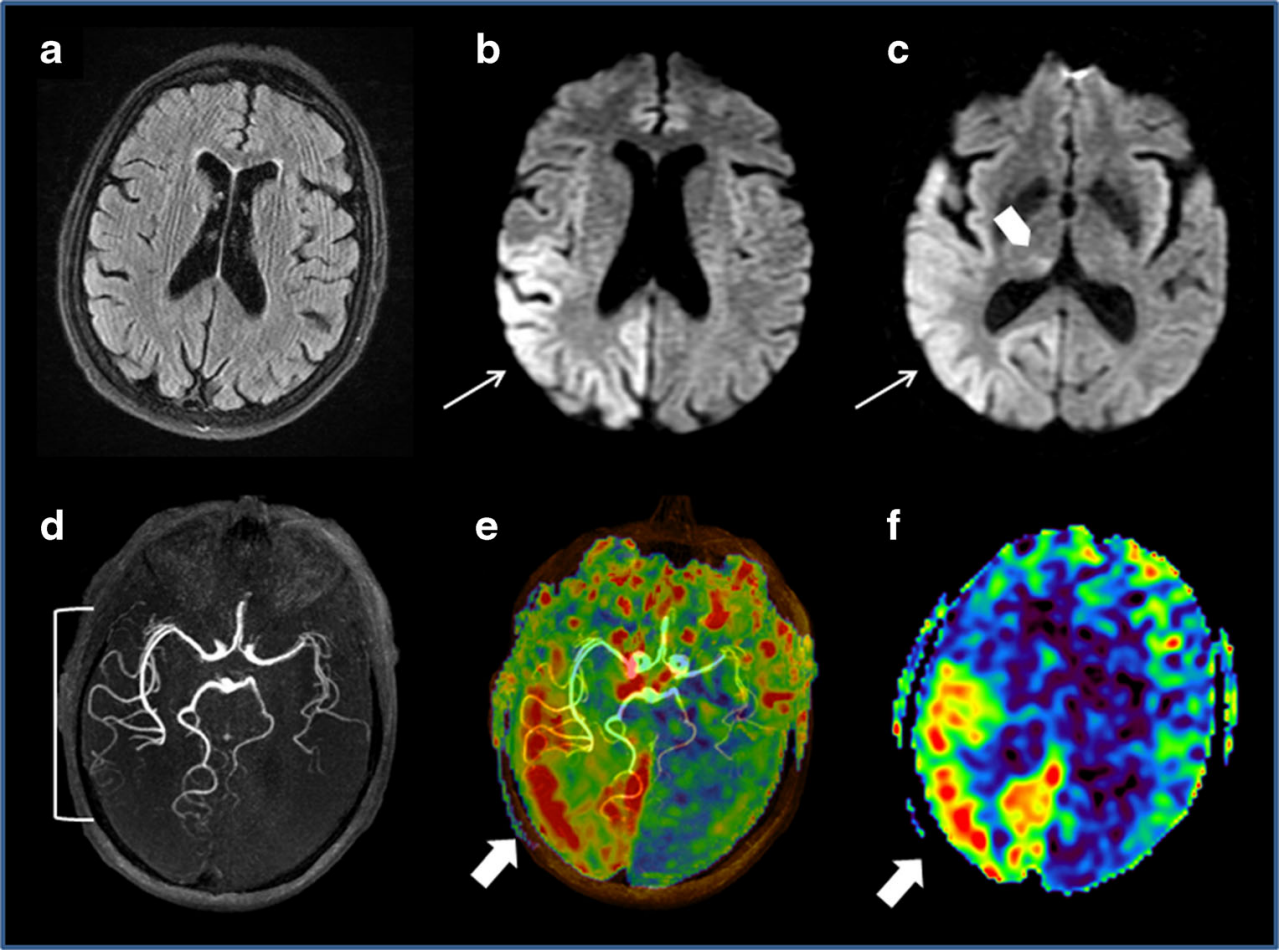
Brain MRI in a 65-year-old patient with sudden-onset left hemibody deficit. FLAIR (**a**), DWI (**b** and **c**), 3D TOF (**d**), ASL/TOF fusion (**e**), and ASL (**f**) sequences in the axial plane. No visible lesion on the FLAIR sequence. **b** and **d** Hyperintense cortical zone on the DWI images showing a right temporo-parieto-occipital zone not corresponding to a vascular territory (*white arrow*), with involvement of the homolateral pulvinar (*arrowhead*). **d**, **e** and **f** Dilatation of the sylvian and right posterior cerebral arteries (*parentheses*) associated with elevated CBF (*white arrows*) in a context of status epilepticus

**Fig. 8:**
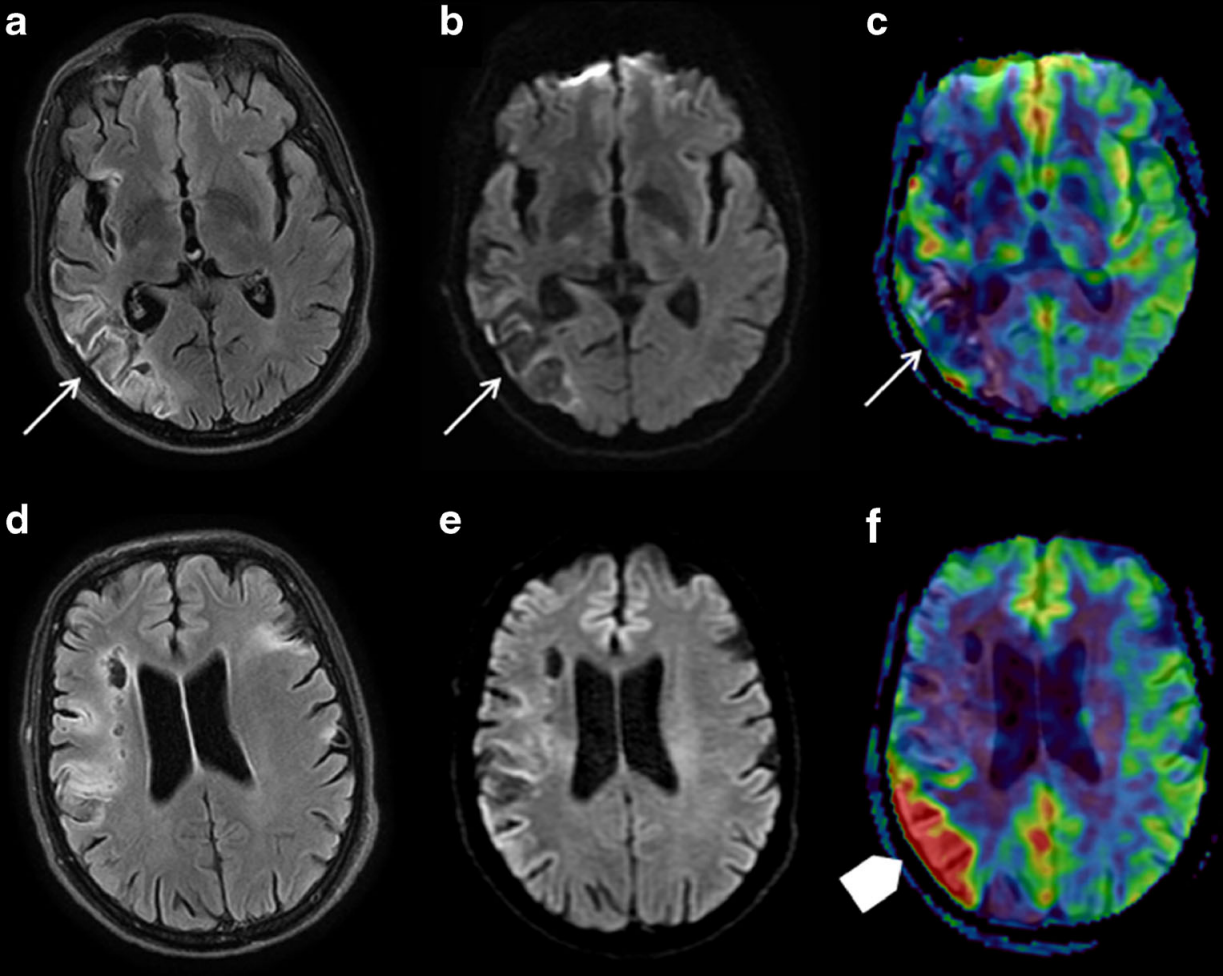
Brain MRI in an 85-year-old patient with a history of right sylvian ischaemic stroke presenting with recurrent left hemibody deficiency. FLAIR (**a** and **d**), DWI (**b** and **e**), and ASL/DWI fusion (**c** and **f**) sequences in the axial plane. **a**, **b**, **c** Sequelar right posterior sylvian zone with no sign of recent ischaemia (*white arrows*). **d**, **e**, **f** Hyperperfusion zone bordering the superior part of the cavity (*arrowhead*), without FLAIR or DWI anomaly, related to a partial seizure on ischaemic sequelae

When there is an epileptic origin, ASL imaging shows focal hyperperfusion during the ictal and early post-ictal phases with increased CBF in the epileptogenic grey matter [50–52]. These zones of hyperperfusion are not limited to a single cerebral vessel territory and are frequently associated with suggestive morphological anomalies such as hypersignals from the pulvinars or the splenium of the corpus callosum on FLAIR and DWI sequences [53] (Fig. 7). ASL can also identify epileptogenic foci, which develop in ischaemic scar tissue in patients given emergency care for suspected recurrent stroke in a previous infarction zone, demonstrating high flow rate zones situated on the borders of parenchymatous sequelae (Fig. 8). In the inter-ictal phase, ASL can identify epileptogenic foci located in focal hypoperfusion zones [54].

**Fig. 9:**
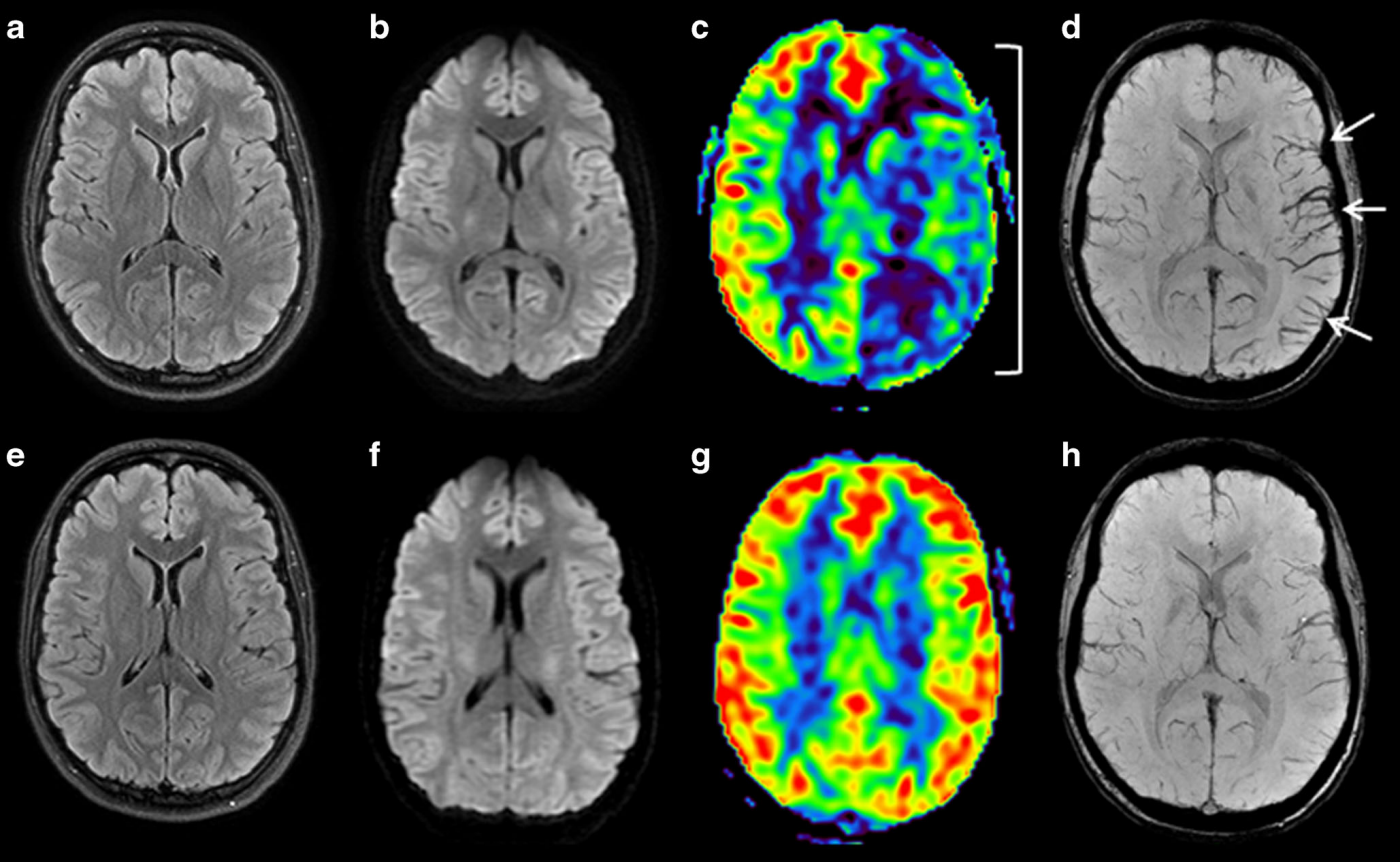
A 21-year-old patient presenting sudden-onset aphasia. Initial (*top row*) and control (*H24, bottom row*) brain MRI with FLAIR (**a**, **e**), DWI (**b**, **f**), ASL (**c**, **g**) and SWI (**d**, **h**) sequences in the axial plane. Initial MRI, **a** and **b** No visible acute ischaemic zone. **c** Large area of hypoperfusion affecting the whole left hemisphere (*parentheses*) related to migraine aura. **d** Left hemispheric brush sign (*white arrows*). Control MRI (H24) shows no ischaemic lesion (**e** and **f**), a normal left hemispheric perfusion (**g**), and a disappearance of the brush sign (**h**)

In migraine aura, perfusion imaging can reveal anomalous focal brain perfusion with a long MTT and decreased CBF and cerebral blood volume [55, 56]. In this situation ASL can also identify areas of decreased CBF [53]. These perfusion anomalies can sometimes resemble those observed in ischaemic stroke and may be associated with a brush sign [57] (Fig. 9), but are frequently bilateral, involving more than a single vascular territory, and predominating in posterior regions [53].

If the imaging is obtained late, at the headache phase, ASL reveals hyperperfusion with increased CBF [53, 58] that can be difficult to distinguish from other stroke mimics such as potential luxury perfusion. Nevertheless, the clinical presentation is usually sufficient to successfully guide diagnosis [53].

## Conclusion

SWI and ASL sequences take advantage of the stronger magnetic field and have demonstrated their contribution to 3-T MRI exploration of acute ischaemic stroke. SWI provides prognostic elements useful for identifying the localisation, morphology, and aetiology of the thrombus, as well as the extent of the parenchymal hypoperfusion, while improving the spatial resolution and sensitivity of T2 gradient-echo sequences for the detection of haemorrhagic transformation. ASL should not be used as a routine sequence because of the acquisition time, but provides precious information for the differential diagnosis in specific situations, giving insight into the early post-therapeutic outcome and a noninvasive assessment of the penumbra.
